# Physical Exercise and Its Protective Effects on Diabetic Cardiomyopathy: What Is the Evidence?

**DOI:** 10.3389/fendo.2018.00729

**Published:** 2018-12-03

**Authors:** Jia Zheng, Jing Cheng, Sheng Zheng, Ling Zhang, Xiaohui Guo, Junqing Zhang, Xinhua Xiao

**Affiliations:** ^1^Key Laboratory of Endocrinology, Department of Endocrinology, Ministry of Health, Peking Union Medical College Hospital, Chinese Academy of Medical Sciences & Peking Union Medical College, Beijing, China; ^2^Department of Endocrinology, Peking University First Hospital, Beijing, China; ^3^The Key Laboratory of Cardiovascular Remodeling and Function Research, Chinese Ministry of Education and Chinese Ministry of Health & The State and Shandong Province Joint Key Laboratory of Translational Cardiovascular Medicine, Shandong University Qilu Hospital, Shandong, China; ^4^Department of Orthopedics, XiangYang Hospital of Traditional Chinese Medicine, Hubei, China

**Keywords:** exercise, diabetic cardiomyopathy, myocardial metabolism, oxidative stress, myocardial fibrosis, apoptosis

## Abstract

As one of the most serious complications of diabetes, diabetic cardiomyopathy (DCM) imposes a huge burden on individuals and society, and represents a major public health problem. It has long been recognized that physical exercise has important health benefits for patients with type 2 diabetes, and regular physical exercise can delay or prevent the complications of diabetes. Current studies show that physical exercise has been regarded as an importantly non-pharmacological treatment for diabetes and DCM, with high efficacy and low adverse events. It can inhibit the pathological processes of myocardial apoptosis, myocardial fibrosis, and myocardial microvascular diseases through improving myocardial metabolism, enhancing the regulation of Ca^2+^, and protecting the function of mitochondria. Eventually, it can alleviate the occurrence and development of diabetic complications. Describing the mechanisms of physical exercise on DCM may provide a new theory for alleviating, or even reversing the development of DCM, and prevent it from developing to heart failure.

## Introduction

The prevalence of type 2 diabetes mellitus (T2DM) continues to increase dramatically, and is now considered as an epidemic worldwide. It is estimated that the number of people with diabetes will exhibit a 45% increase in three decades (1). Uncontrolled diabetes can lead to a number of long-term health complications, including heart diseases, nerve damage, vision problems, and amputation. Among the various complications of diabetes, cardiovascular disease (CVD) represents the major cause of mortality and morbidity in diabetic patients, accounting for nearly 70% of related heart failure cases (2). Diabetic patients, when compared with non-disease subjects, are two to four times more likely to experience CVD events, due to micro- and macrovascular atherosclerosis, which is often exacerbated by the presence of concomitant CVD risk factors, including hypertension, dyslipidemia, and activation of neuro-hormonal and inflammatory mechanisms (3, 4).

## The pathogenesis of DCM

Diabetic cardiomyopathy (DCM), a distinct condition that develops in diabetic patients, is defined by the presence of myocardial dysfunction in the absence of coronary atherosclerosis, overt clinical coronary artery disease (CAD), and valvular heart disease (5, 6). It is characterized by cardiac structure and function disorders, including myocardial fibrosis, dysfunctional remodeling, and associated metabolic deregulation and left ventricular dysfunction (7–9). DCM is associated with diastolic dysfunction, with depressed myocardial contractility and relaxation, and eventually by clinical heart failure (10). Left ventricular diastolic dysfunction with increased wall stiffness is common in subjects with well-controlled T2DM who are free of clinically detectable heart diseases (11). The pathogenesis of DCM is a multifactorial process that includes altered myocardial metabolism (impaired energy metabolism, calcium regulation, and mitochondrial function) (12, 13), increased oxidative stress (14), altered myocardial structure with fibrosis (15), higher induction of apoptosis (16), and microvascular disease (9).

## Current strategies for DCM intervention

Despite prominent advances in the prevention and treatment of diabetes, diabetic complications, especially for DCM still remain rigorous in patients with T2DM (17, 18). Nowadays, treatments for DCM include glucose and lipid control, hypertension treatment, and CAD intervention. Currently, pharmacological treatment is accepted as the common strategy for CVD in diabetic patients, such as β-blockers, Ca^2+^ antagonist, β-blockers, renin–angiotensin–aldosterone system inhibitors (9). In recent years, it demonstrated that metformin and sodium-dependent glucose transporters 2 (SGLT2) inhibitors are also benefit for DCM (19). A systematic review of observational studies showed that metformin reduced all-cause mortality in patients with diabetes with congestive heart failure (20). Pan et al. found that empagliflozin ameliorated DCM in diabetic ALDH2^*^2 mutant patients (21). However, the incidence and mortality rate of DCM still remains high. Physical exercise has been shown to improve health and quality of life in patients with a variety of diseases, including obesity (22), T2DM (23), chronic kidney disease (24), and cancers (25). Currently, physical exercise has been regarded as an importantly non-pharmacological treatment for the prevention and treatment of diabetes and its complications. It has been long known that physical activity can decrease the occurrence of cardiac events, including heart attacks and strokes, and the need for a coronary revascularization intervention (26–28). Exercise induces adaptations to the heart itself, as well as the cardiovascular system. These adaptations include protection against ischemic damage, increase of cardiac growth, and modulation of cardiac metabolism, function, and vascular supply (26). Myocardial apoptosis, myocardial fibrosis, and hemodynamic disorders caused by high glucose can be improved or even reversed by physical exercise. Studies have shown that exercise can improve myocardial metabolism, lower blood glucose, increase insulin sensitivity, inhibit myocardial fibrosis, improve oxidative stress, and decrease the risks of CVDs. Ultimately, it can improve heart function and decrease the mortality of DCM (29, 30).

## Clinical studies about exercise and diabetes-related cardiovascular diseases

Physical activity plays an essential role in the maintenance of human health. Chronic diseases, such as metabolic syndrome and diabetes, are a tremendous burden to our society. Regular physical activity is a primary recommendation for the prevention and treatment of these diseases (36). Physical exercise has a wide array of beneficial effects, including improving glucose and insulin metabolism, and reducing the risks of CVDs in diabetic patients (37). The intensive weight loss intervention was effective in increasing physical activity and improving cardiorespiratory fitness in overweight and obese individuals with T2DM (31). Physical activity was associated with reduced risks of CVD, cardiovascular death, and total mortality in patients with T2DM (32, 38). In addition, the benefits of exercise may depend on the intensity of exercise. It showed that a moderate to high level of physical fitness was independently associated with several cardiovascular risk markers, which may contribute to decreasing the burden of morbidity and mortality in patients with T2DM (33). Hu et al. also showed that a moderate or high level of physical activity was associated with a reduced risk of total and cardiovascular mortality among patients with T2DM, which was regardless of the levels of body mass index, blood pressure, total cholesterol, and smoking (34). Furthermore, the duration of exercise is also an important factor for its benefits of CVD. Karjalainen et al. found that there was an inverse association between leisure-time physical activity and short-term CVD outcome. However, controlled, home-based exercise training had minor effects on the risk profile of CVD in CAD patients with T2DM. These findings highlight the significance of lifelong physical activity instead of a short-term exercise program in the prevention of future unfavorable outcomes in patients with CAD (35). However, clinical studies about exercise and its benefits in diabetes-related CVDs are limited. It is suggested to carry out long-term exercise program to ensure maximum exercise efficiency, with an appropriate amount of exercise. According to the evidence of the aforementioned studies and the 2018 guideline of American Diabetes Association (ADA) (39), we recommend a moderate to high level [≥4 to <7 metabolic equivalents (METs)] of physical fitness for patients with T2DM, such as 150 min or more of moderate-to-vigorous intensity aerobic activity per week. The clinical studies about exercise and diabetes-related cardiovascular diseases are listed in Table 1.

**Table 1:** Clinical studies about exercise and diabetes-related cardiovascular diseases.

**Subjects included**	**Exercise profile**	**Metabolic effects**	**References**
4,376 overweight or obese adults with T2DM	At least 50 min/week, progressing to at least 175 min/week	Increased physical activity Improved cardiorespiratory fitness	Jakicic et al. (31)
3,058 patients with T2DM	Leisure-time physical activity	Reduced risk of CVD, cardiovascular death, and total mortality	Tanasescu et al. (32)
575 patients with T2DM	Low (<4 METs), moderate (≥4 to <7 METs) and high fitness (≥7 METs)	Lower hemoglobin A1c and C-reactive protein levels A decreased prevalence of left ventricular hypertrophy and increased aortic stiffness	Cardoso et al. (33)
3,708 patients with T2DM	Occupational, commuting, and leisure-time physical activity	Decreased total and cardiovascular mortality	Hu et al. (34)
539 patients with T2DM	Leisure-time physical activity	Reduced risks of short-term CVD outcome	Karjalainen et al. (35)

*CVD, cardiovascular disease; METs, metabolic equivalents*.

## Pre-clinical experiments about physical exercise and DCM

### Physical exercise improves cardiomyocyte metabolism

#### Physical exercise increases energy metabolism

Disorders of myocardial glucose and lipid metabolism lead to changes in pathways related with myocardial energy metabolism. Abnormalities that produce cardiac structure and function are called “metabolic remodeling of the heart,” which ultimately leads to the development of cardiomyopathy. Glucose transporter-4 (GLUT-4) is an intracellular protein that can be translocated to cell membrane induced by insulin, and then it can participate in glucose uptake and utilization. The expression of GLUT-4 was decreased and abnormally distributed in diabetic state, resulting in a significant decrease in glucose transport and impaired myocardium energy utilization (58). Studies indicate that moderate exercise can upregulate GLUT-4 expression, and also can increase glucose transport and activate pyruvate dehydrogenase complexes, even in the absence of insulin (59). It suggests that exercise can compensate for impaired energy metabolism in insulin-deficient state, which may be related to the increase of insulin-sensitive adenosine monophosphate activated protein kinase (AMPK) expression, thereby protecting pancreatic β cells. Exercise may also enhance insulin-mediated glucose transport by increasing the expression of protein kinase C-δ (60, 61). Exercise can also increase insulin and its downstream protein expressions in the myocardium of diet-induced obesity rats, as well as forkhead box protein o1 (Foxo1) and other key regulators of pancreatic β cells, and also activate insulin signaling pathway (40). Thus, exercise can protect pancreatic β cells, promote insulin secretion, activate insulin signaling pathway, increase GLUT4 expression, improve intracellular energy metabolism, and ultimately protect cardiomyocytes.

#### Physical exercise enhances calcium regulation

Calcium is a crucial mediator of cell signaling in skeletal muscles for cellular functions and specific functions, including contraction, fiber-type differentiation, and energy production. Intracellular Ca^2+^ dyshomeostasis is one of the main markers of DCM, which can affect myocardial contractile function, directly leading to the occurrence and development of DCM. It is even worse in altered sarcoplasmic reticulum Ca^2+^ uptake rate accompanied by decreased function of sarcoplasmic reticulum Ca2+-ATPase (SERCA2a) (62). In T2DM patients, the Na^+^-Ca^2+^ exchange of cardiomyocytes is inhibited, while the sarcoplasmic reticulum Ca^2+^ pump is normal, and Ca^2+^ is gradually concentrated in the sarcoplasmic reticulum. Thus, the amplitude and attenuation rate of Ca^2+^ concentration in the myocardium is decreased. Conversely, exercise can improve the expression and activity of SERCA2a, which can regulate Ca^2+^ release and recapture in the myocardium. Exercise can increase Ca^2+^-calmodulin-dependent protein kinase phosphorylation, reduce Ca^2+^ efflux, facilitate Ca^2+^ regulation, and ultimately improves myocardial contraction and diastolic function (41). Stølen et al. found that high intensity intermittent exercise improved myocardial contractility by restoring L-type Ca^2+^ channels, increasing the density of T-transverse tubules, and increasing the synchrony of Ca^2+^ release and excitatory contraction coupling (42).

#### Physical exercise improves mitochondrial function

Mitochondrion is the center of energy metabolism, and recent evidence suggests that mitochondrial dysfunction may play a critical role in the pathogenesis of DCM. The imbalance of energy supply and demand directly leads to the decline of myocardial function and induction of DCM (63). The ultrastructure of mitochondria in DCM shows reduced density, mitochondrial swelling, and destruction of the intima and adventitia, and an increase in mitochondrial matrix, while exercise attenuates diabetes-induced ultrastructural changes in rat cardiac tissue (43). Moderate exercise intervention has a protective effect on mitochondrial function. Exercise can regulate the key regulator of mitochondrial metabolism, peroxisome proliferator-activated receptor gamma co-stimulatory factor-1α (PGC-1α), and activate its downstream transcription factors. Thus, it can enhance mitochondrial DNA replication and transcription, and increase mitochondria biosynthesis (44). Furthermore, the mechanisms by which exercise improves mitochondrial function may be related to the regulation of Ca^2+^ in mitochondria. Ca^2+^ is a key metabolic enzyme activator in mitochondria, and mitochondrial Ca^2+^ circulatory balance can be easily affected by intracellular Ca^2+^ homeostasis (41, 42). Resistance exercise improves cardiac function and mitochondrial efficiency in hearts, of diabetic rat, which were accompanied by higher expressions of mitochondrial biogenesis proteins such as PGC-1α and mitochondrial transcription factor A (TFAM) (45). In addition, studies have shown that high intensity exercise can increase myocardial mitochondrial contents, but no change in moderate intensity exercise (46, 47). However, Veeranki et al. showed that moderate intensity exercise prevented DCM associated contractile dysfunction through restoration of mitochondrial function and connexin 43 levels in db/db mice (30). These indicate that myocardial mitochondrial biosynthesis may be associated with exercise intensity, and exercise intensity should be further investigated about its effects on DCM.

### Physical exercise relieves oxidative stress damage

Oxidative stress is considered to be a key link in the development of DCM. Under physiological conditions, there is a balance system of oxygen free radicals and free radicals in the body. Oxygen atoms play an important role in the redox signaling pathway. Moderate oxidation can increase protein activity, but excessive reactive oxygen species can cause pathological changes through interaction with lipids, proteins, and DNA (64). Hyperglycemia can directly promote the production of oxygen free radicals, induce oxidative stress, and promote cardiomyocyte apoptosis. The mechanisms by which exercise ameliorates oxidative stress is complex, including: (1) reducing the production of reactive oxygen species. Exercise can ameliorate the damage caused by excessive oxidative stress in the diabetic myocardium and pancreas, thereby improving glucose metabolism and reducing damage caused by reactive oxygen species (48). Long-term exercise can also directly reduce the level of reactive oxygen species in the body by reducing the activity of nicotinamide adenine dinucleotide phosphate (NADPH) oxidase in diabetic rats (49). (2) Enhancing the ability of anti-oxidative stress. Exercise can increase the expression of nitric oxide synthase and nitric oxide, and ultimately enhance the antioxidant function in endothelial cells (49). Nuclear factor E2-related factor 2 (Nrf2) can regulate the expressions of antioxidants mediated by antioxidant response elements. It is an important transcription factor for intracellular defense of reactive oxygen species (50, 65). Studies have shown that acute exercise can promote the function of Nrf2, activate downstream antioxidant response elements, and ultimately enhance the activity of anti-oxidative stress. In addition, knocking out the Nrf2 gene can increase the sensitivity of cardiomyocytes to oxidative stress, leading to increased oxidative damage in cells (50). Kanter et al. showed that low intensity exercise decreased the elevated tissue malondialdehyde (MDA) levels and increased the reduced activities of the enzymatic antioxidants superoxide dismutase (SOD), glutathione peroxidase (GSH-Px), and catalase (CAT) in cardiac tissue (51). It indicates that exercise improves the biological mechanisms of DCM by affecting the levels of plasminogen activator inhibitor 1 (PAI-1) and endothelial nitric oxide synthase (eNOS), and it is dependent on the intensity of exercise (52).

### Physical exercise improves myocardial fibrosis

Myocardial fibrosis is the most prominent histopathological change in DCM, characterized by myocardial cell collagen deposition, interstitial fibrosis, and perivascular fibrosis, and ultimately induce the reconstruction of cardiac structure and function (53). Numerous studies indicated that moderate exercise can decrease blood glucose, reduce myocardial fibrosis, promote myocardial reverse remodeling in diabetic rats, and improve cardiac function (30, 53). The mechanisms may be that exercise reduces pressure overload by improving blood pressure, thereby alleviating myocardial fibrosis (53). Exercise can increase the content of matrix metalloproteinase-2 (Mmp-2) in obese rats, increase the degradation of collagen and inhibit the formation of myocardial fibrosis (47). The interaction of collagen with glucose can further cause chemical modification of glycated collagen to form advanced glycation end products (AGEs) that promote arterial and cardiac cirrhosis, as well as endothelial dysfunction (66). Other mechanism by which exercise improves myocardial fibrosis may be related to improving energy metabolism, decreasing blood glucose, and myocardial glycogen deposition (54). Novoa et al. showed that high intensity chronic exercise had a positive impact on cardiac remodeling, evidenced as reduction in myocyte hypertrophy, reduced collagen deposition, and amelioration of myocardial fibrosis (55).

### Physical exercise inhibits cardiomyocyte apoptosis

Diabetes-induced cardiomyocyte apoptosis is a typical feature of DCM. Hyperglycemia can directly promote cytochrome C release to the cytoplasm by activating cytochrome C in mitochondria, triggering cascade activation of caspase-3, leading to endogenous apoptosis of cardiomyocytes. This change plays an important role in the development of diabetic cardiac hypertrophy, myocardial remodeling, and heart failure. C-Jun N-terminal kinase is a member of the mitogen-activated protein kinase (MAPK) family, which can activate caspase-8 and the apoptotic protein Bax, and release cytochromes to promote apoptosis (67). Veeranki et al. found that exercise can also reduce cytochrome C leakage into cytoplasm by increasing mitochondrial transmembrane potential, thus prevent cardiomyocyte apoptosis (30). A number of studies have shown that exercise can reduce the phosphorylation of c-Jun N-terminal kinase in obese rats, block the transmission of downstream apoptotic signals. Exercise can also increase the expression of B-cell lymphokine 2 in the myocardium of diabetic mice, which can affect the activation of pro-apoptotic proteins by binding to pro-apoptotic proteins, and ultimately play an anti-apoptotic role on cardiomyocytes in diabetic mice (56). Kanter et al. showed that low intensity exercise had a therapeutic effect on diabetes-induced morphological, biochemical, and apoptotic changes in the cardiac tissue of rats (51). Khakdan et al. found that high intensity interval training effectively increased the expression of Sirtuin 1 (Sirt1) and B cell leukemia/lymphoma 2 (BCL-2) in diabetic rats, with improved left ventricular ejection fraction (LVEF%) and fractional shortening (FS%) (57). A recent study suggested that exercise appeared to ameliorate DCM by inhibiting endoplasmic reticulum stress-induced apoptosis in diabetic rats, which was in an intensity-dependent manner (52).

### Physical exercise improves microvascular disorders

**Figure 1:**
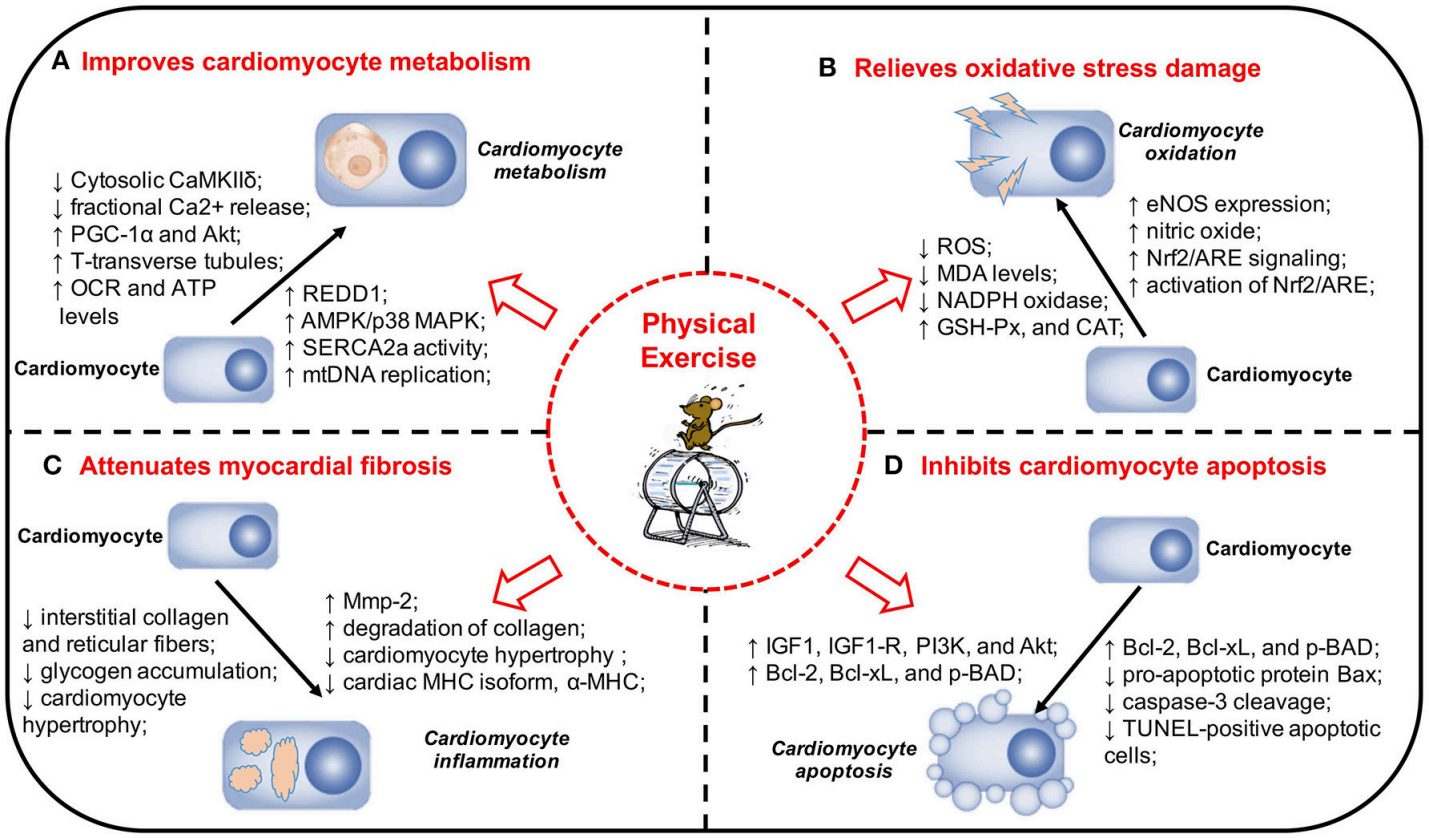
Physical exercise and its protective effects and potential mechanisms in DCM. **(A)** Physical exercise Improves cardiomyocyte metabolism in DCM. **(B)** Physical exercise relieves oxidative stress damage in DCM. **(C)** Physical exercise attenuates myocardial fibrosis in DCM. **(D)** Physical exercise inhibits cardiomyocyte apoptosis in DCM. The pathogenesis of DCM is a multifactorial process and a range of mechanisms play a significant role in the onset and development of DCM. Physical exercise can exert a variety of protective effects on DCM, including improving cardiomyocyte metabolism, relieving oxidative stress damage, attenuating myocardial fibrosis, and inhibiting cardiomyocyte apoptosis. DCM, diabetic cardiomyopathy; MAPK, mitogen-activated protein kinase; REDD1, regulated in development and DNA damage response 1; SERCA2a, sarcoplasmic reticulum Ca2+-ATPase; mtDNA, mitochondrial DNA; PGC-1α, Peroxisome proliferator-activated receptor gamma coactivator 1-alpha; Akt, protein kinase B; VO2max, maximal oxygen consumption; EF, ejection fraction; FS, fractional shortening; OCR, oxygen consumption rate; ATP, adenosine triphosphate; eNOS, endothelial nitric oxide synthase; NADPH, nicotinamide adenine dinucleotide phosphate; Nrf2, nuclear factor erythroid 2-related factor 2; ARE, antioxidant responsive element; MDA, malondialdehyde; SOD, superoxide dismutase; GSH-Px, glutathione peroxidase; CAT, catalase; MHC, myosin heavy chain; Mmp-2, matrix metalloproteinase-2; IGF1, insulin-like growth factor 1; IGF1-R, IGF1-receptor; PI3K, phosphatidylinositol 3′-kinase; TUNEL, TdT-mediated dUTP Nick-End Labeling.

Microvascular disease is also one of the pathological changes of DCM. Under the influence of hyperglycemia, the function and structure of microvessels will undergo pathological changes, which are characterized by vascular endothelial defects, endothelial cell dysfunction, and aggravated inflammatory response of partial vascular endothelium. It can affect the transport of substances, such as glucose and insulin into other tissues of the body, which can lead to abnormal tissue function. The mechanisms underlying the protective effects of exercise on microvessels mainly include two aspects: (1) exercise can protect vascular endothelial cells, increase the expression of nitric oxide, enhance the diastolic function of microvessels, and increase blood perfusion (66). (2) Exercise can enhance microvascular response to insulin and improve insulin signaling. Increased insulin can activate both insulin receptor substrate-1/phosphatidylinositol-3-kinase/protein kinase B (IRS-1/PI3K/AKT) and MAPK pathways, leading to equilibrium between the vasopressor substance nitric oxide and endothelin-1, ensuring normal vasomotor function (68). The pre-clinical experiments and the potential mechanisms about physical exercise and DCM is shown in Table 2 and Figure 1.

**Table 2:** Pre-clinical experiments about physical exercise and DCM.

**Animals**	**Exercise intervention**	**Main findings**	**References**
**PHYSICAL EXERCISE IMPROVES CARDIOMYOCYTE METABOLISM**			
Diet-induced obesity rats	Treadmill running (50-min/day, 5 days per week velocity of 1.0 km/h for 2 months)	- Increased protein levels of phospho-P38MAPK, REDD1 in the myocardium - Decreased 14-3-3 protein levels in the myocardium	Pieri et al. (40)
STZ-induced diabetic SD rats	accumulated about 3,554 m/day of voluntary wheel running for 12 weeks	- Prevented diastolic dysfunction in diabetic mice - Normalized sarcoplasmic reticulum protein content and expression in diabetic animals - Enhanced SERCA2a activity	Epp et al. (41)
Cardiomyocytes from mice with T2DM (db/db)	13 weeks of aerobic interval training (4 min at 85–90% of VO_2max_ and 2 min at 50% of VO_2max_ for 80 min /day, 5 days/week)	- Restored contractile function associated with restored SR Ca^2+^ release synchronicity, T-tubule density, twitch Ca^2+^ amplitude, SR Ca^2+^ ATPase and Na^+^/Ca^2+^-exchanger activities, and SR-Ca^2+^ leak - Reduced phosphorylation of cytosolic CaMKIIδ - Normalized enhanced fractional Ca^2+^ release	Stølen et al. (42)
STZ-induced diabetic SD rats	Run daily on a treadmill for 9 weeks (60 min/day, at a pace of 20 m/min)	- Attenuated diabetes-induced changes in collagen fibrils, cytoplasmic area, and level of mitochondrial disruption	Searls et al. (43)
C57BL/6 db/db mice	Run daily on a treadmill for 15 weeks (10 m/min for 1 h/day)	- Reversed reduction in EF and FS - Reversed reduction of mtDNA replication and transcription, together with reduced mtDNA content and impaired mitochondrial ultrastructure - Activated PGC-1α and Akt signaling	Wang et al. (44)
Otsuka Long-Evans Tokushima Fatty rats	20 repetitions of climbing a ladder 5 days per week for 12 weeks	- Increased EF and FS - Increased mitochondrial numbers - Higher expression of PGC-1α and TFAM	Ko et al. (45)
C57BL/6J mice	10 weeks of treadmill running (4 min at 85–90% of VO_2max_)	10% increase in heart weight-to-body weight ratio 36% increase in glucose oxidation and a concomitant reduction in fatty acid oxidation	Hafstad et al. (46)
Diet-induced obesity C57BL/6 mice	8–10 weeks of treadmill running (4 min at 85–90% of VO_2max_)	- Improved aerobic capacity, reduced obesity, improved glucose tolerance - Normalized left ventricular mechanical efficiency and mechanoenergetics - Improved mitochondrial capacity and efficiency, as well as reduced oxidative stress	Hafstad et al. (47)
db/db mice	300 m run on a treadmill for 5 days/week at the speeds of 10–11 m/min for 5 weeks	- Prevented diabetic cardiac functional deficiencies: EF and FS - Improvements in contraction velocity and contraction maximum, OCR, and tissue ATP levels - Attenuated transmembrane potential decline and cytochrome c leakage	Veeranki et al. (30)
**PHYSICAL EXERCISE RELIEVES OXIDATIVE STRESS DAMAGE**			
STZ-induced diabetic Wistar rats	9 weeks of treadmill running (11 m/min, 18 min/day)	- Lower left atrium diameter - Higher catalase and superoxide dismutase activities - Higher glutathione peroxidase activity	Gimenes et al. (48)
Diabetic Goto-Kakizaki (GK) rats	9 weeks of treadmill running (60 min/day and 5 days/week)	- Increased plantaris muscle cytochrome oxidase, improved glycosylated hemoglobin and insulin sensitivity - Increased both total eNOS expression and the dimer:monomer ratio in the left ventricle - Increased nitric oxide (+28%) production and decreased eNOS-dependent superoxide (−12%) production Decreased NADPH-dependent O_2_-activity	Grijalva et al. (49)
Nrf2^−/−^ mice	Exercise on a treadmill for 2 consecutive days (60 min/day; 14 m/min; 10% grade)	- Activated Nrf2/ARE signaling and promoted antioxidant - Activation of Nrf2/ARE (antioxidant response element) signaling - Enhancement of antioxidant defense pathways - Increased trans-activation of ARE-containing genes	Muthusamy et al. (50)
STZ-induced diabetic SD rats	Exercise on a treadmill for 30 min daily for 4 weeks at a speed of 10 m/min	- Decreased the elevated tissue MDA levels - Increased the reduced activities of the enzymatic antioxidants SOD, GSH-Px, and CAT in cardiac tissue	Kanter et al. (51)
STZ-induced diabetic SD rats	Exercise on a treadmill for 60 min/day on 5 days for 6 weeks (10–20 m/min)	- Higher serum level of NO and eNOS - Reduced PAI-1 and vWF - Reduced PKC levels	Chengji et al. (52)
**PHYSICAL EXERCISE ATTENUATES MYOCARDIAL FIBROSIS**			
db/db mice	300 m run on a treadmill for 5 days/week at the speeds of 10–11 m/min for 5 weeks	- Normalized overall collagen accumulation at both the perivascular regions and in interstitial regions of heart tissue - Prevented the tendency for decline in the fast twitch cardiac MHC isoform, α-MHC	Veeranki et al. (30)
STZ-induced diabetic Wistar rats	Swimming training for 8 weeks (5 days/week, 90 min/day, with a load of 5% body weight)	- Decreased interstitial collagen and reticular fibers on the extracellular matrix - Attenuated glycogen accumulation	Silva et al. (53)
Diet-induced obesity C57BL/6 mice	8–10 weeks of treadmill running (4 min at 85–90% of VO_2max_)	- Increased the content of Mmp-2 in obese rats, increase the degradation of collagen and inhibited the formation of myocardial fibrosis	Hafstad et al. (47)
High-fat diet fed C57BL/6J mice	5 weekly HIT (10 × 4 min at 85–90% of maximum oxygen uptake)	- Normalized diastolic function, attenuated diet-induced changes in myocardial substrate utilization - Inhibited cardiac reactive oxygen species content and fibrosis	Lund et al. (54)
Alloxan-induced diabetic SD rats	Exercise on a treadmill for 4 weeks at 80% of maximal performance	- Inhibited cardiomyocyte hypertrophy - Inhibited collagen deposition in the heart and interstitial fibrosis	Novoa et al. (55)
**PHYSICAL EXERCISE INHIBITS CARDIOMYOCYTE APOPTOSIS**			
db/db mice	300 m run on a treadmill for 5 days/week at the speeds of 10–11 m/min for 5 weeks	- Attenuated transmembrane potential decline and cytochrome c leakage	Veeranki etal. (30)
STZ-induced diabetic Wistar rats	Exercise on a treadmill for 60 min/day, 5 days/week, for 10 weeks	- Increased cardiac survival pathway (IGF1, IGF1-R, PI3K, and Akt) and the pro-survival Bcl-2 family proteins (Bcl-2, Bcl-xL, and p-BAD) - Reduced cardiac TUNEL-positive apoptotic cells - Decreased the apoptotic key component caspase-3	Cheng et al. (56)
STZ-induced diabetic SD rats	Exercise on a treadmill for 30 min daily for 4 weeks at a speed of 10 m/min	- Reduced cardiac TUNEL-positive apoptotic cells	Kanter et al. (51)
High-fat high-fructose diet-induced Wistar diabetic rats	Exercise on a treadmill for 5-min at 30–40% of VO_2_max, 2-min intervals at 85–90% VO_2_max with recovery cycles at 30–40% VO_2_max and finished by 3-min cooling down by running at 30–40% of VO_2_max for 10 weeks	- Increased the expression of Sirt1 and BCL-2 - Increases LVEF% and FS%	Khakdan et al. (57)
STZ-induced diabetic SD rats	Exercise on a treadmill for 60 min/day on 5 days for 12 weeks (20 m/min for LIT and 34 m/min for HIT)	- Reduced serum cTn-I levels - Reduced GRP78, CHOP, and cleaved caspase-12 protein expression	Chengjier et al. (52)

*DCM, diabetic cardiomyopathy; STZ, Streptozotocin; SD, Sprague-Dawley; MAPK, mitogen-activated protein kinase; REDD1, regulated in development and DNA damage response 1; SR, sarcoplasmic reticulum; SERCA2a, sarcoplasmic reticulum Ca^2+^-ATPase; mtDNA, mitochondrial DNA; PGC-1α, Peroxisome proliferator-activated receptor gamma coactivator 1-alpha; TFAM, mitochondrial transcription factor A; Akt, protein kinase B; VO2max, maximal oxygen consumption; LVEF, left ventricular ejection fraction; EF, ejection fraction; FS, fractional shortening; OCR, oxygen consumption rate; ATP, adenosine triphosphate; PAI-1, plasminogen activator inhibitor 1; vWF, Von Willebrand factor; PKC, protein kinase C; NO, nitric oxide; eNOS, endothelial nitric oxide synthase; NADPH, nicotinamide adenine dinucleotide phosphate; Nrf2, nuclear factor erythroid 2-related factor 2; ARE, antioxidant responsive element; MDA, malondialdehyde; SOD, superoxide dismutase; GSH-Px, glutathione peroxidase; CAT, catalase; MHC, myosin heavy chain; Mmp-2, matrix metalloproteinase-2; HIT, high intensity interval training; IGF1, insulin-like growth factor 1; IGF1-R, IGF1-receptor; PI3K, phosphatidylinositol 3′-kinase; TUNEL, TdT-mediated dUTP nick-end labeling; LIT, low intensity exercise training; cTn-I, cardiac troponin I; Sirt1, Sirtuin 1; BCL-2, B cell leukemia/lymphoma 2; GRP78, glucose-regulated protein 78; CHOP, C/EBP homologous protein*.

## Conclusions

In summary, as a fundamental component of the human condition, physical exercise plays a critical role in human health. Exercise training is considered as a cornerstone in the management of T2DM, possessing a potency to decrease the risks of CVD in patients with diabetes. Exercise can protect the myocardium by improving myocardial cell metabolism, alleviating oxidative stress damage, improving myocardial fibrosis, inhibiting apoptosis, and ameliorating microvascular disorders, and ultimately it is proposed to have the potential impacts to protect against DCM. Exercise is an importantly non-pharmacological strategy in reducing the risk factors of diabetes and its complications. It can be considered as a promising agent for alternative therapies for the prevention and treatment of diabetes and its cardiovascular complications. However, more clinical trials and pre-clinical studies are required to promote the translation of molecular findings to therapeutics of physical exercise.

## Author contributions

JZ and JC collected data, synthesized data, and wrote the manuscript. SZ, LZ, and XHG reviewed and edited the manuscript. JQZ and XHX contributed to the design of this review.

### Conflict of interest statement

The authors declare that the research was conducted in the absence of any commercial or financial relationships that could be construed as a potential conflict of interest.
